# In vitro hepatotoxicity evaluation of methotrexate-loaded niosome formulation: fabrication, characterization and cell culture studies

**DOI:** 10.55730/1300-0144.5651

**Published:** 2023-03-07

**Authors:** Ahmet Doğan ERGİN, Çağatay OLTULU, Nebiye Pelin TÜRKER, Gülen Melike DEMİRBOLAT

**Affiliations:** 1 Department of Neuroscience, University of Torino, Torino, Italy; 2 Department of Pharmaceutical Technology, Faculty of Pharmacy, Trakya University, Edirne, Turkiye; 3 Department of Pharmaceutical Toxicology, Faculty of Pharmacy, Trakya University, Edirne, Turkiye; 4 Technology Research Development Application and Research Center, Trakya University, Edirne, Turkiye; 5 Department of Pharmaceutical Technology, Faculty of Pharmacy, Acıbadem Mehmet Ali Aydınlar University, İstanbul, Turkiye

**Keywords:** Methotrexate, niosomes, hepatotoxicity, oxidative stress, apoptosis

## Abstract

**Background/aim:**

Methotrexate (MTX) is a folic acid antagonist that is widely used to treat osteosarcoma, leukemia, breast cancer, and autoimmune and inflammatory diseases. The most important concerns with MTX are its poor solubility and high toxicity, particularly in liver cells. To enhance its solubility and to minimize its toxicity, we encapsulated MTX in niosomes and investigated its hepatotoxicity mechanisms using genetic biomarkers.

**Materials and methods:**

Niosomes were successfully prepared using a modified thin film method, and the prepared monodisperse small-sized formulation was subsequently characterized. In vitro cytotoxicity studies were performed both in hepatocarcinoma (HEP3G) and healthy liver (AML12) cell lines. Specifically, immunofluorescence assay and evaluation of the expression levels of apoptotic, antioxidant, heat shock protein, and oxidative stress genes were performed.

**Results:**

The formulation had a particle size of 117.1 ± 33 nm, a surface charge of −38.41 ± 0.7 mV, and an encapsulation efficiency of 59.7% ± 2.3%. The results showed that the niosomal formulation exhibited significantly higher cytotoxic effects in HEP3G than in AML12. The immunofluorescence and genetic analyses showed that the increased cytotoxicity of niosomes resulted mainly from oxidative stress and slight apoptosis.

**Conclusion:**

These results demonstrated that niosomal drug delivery systems could be a new potential formulation for minimizing MTX-related hepatotoxicity.

## 1. Introduction

Methotrexate (2,4-diamino-N10-methyl propylglutamic corrosive, MTX), an antimetabolite of folic acid is used as an anticancer drug for acute lymphoblastic leukemia, bladder cancer, breast cancer, non-Hodgkin lymphomas, and osteosarcoma. Additionally, it is used to treat autoimmune and inflammation-related conditions such as Crohn’s disease, multiple sclerosis, and psoriasis, rheumatoid arthritis [1, 2]. MTX is a weak acid with poor permeability (C logP = 0.53), low aqueous solubility (0.01 mg/mL) [3]. Also, the bioavailability of MTX is dose dependent [4].

The major side effects of MTX are bone marrow depression and hepatotoxicity. MTX exerts its essential toxic effects against the rapidly duplicating cells of the bone marrow and gastrointestinal epithelium, and these cells cause leukopenia and thrombocytopenia. MTX-related hepatotoxicity could result from direct damage to hepatocytes and/or from concomitant hepatitis. Both low- and high-dose MTX treatments can lead to hepatotoxicity [5–7]. However, the exact mechanism of MTX-induced liver injury remains unknown. Recent reports have suggested that hepatotoxicity is the result mainly of oxidative stress, which decreases methionine synthesis, leading to insufficient production of antioxidants (e.g., glutathione, superoxide dismutase, and catalase), increased reactive oxygen species (ROS), mitochondrial degeneration, and microvascular steatosis [5].

We previously developed MTX-loaded niosomes, and we evaluated them in terms of efficacy in different cancer cells [8]. In the present study, the MTX-loaded niosome formulations we previously developed were used to reduce the hepatotoxicity of MTX. The prepared niosomes were characterized by several techniques, such as FTIR, SEM, dynamic light scattering (DLS) and zeta potential. The cytotoxic effects of pure MTX and MTX-loaded niosomes were evaluated in cancer (HEP3B) and healthy (AML12) liver cell cultures. The cell death mechanisms of pure MTX and MTX-loaded niosomes at predetermined doses were investigated by an immunofluorescent assay. To investigate the genetic mechanisms in detail, we analyzed pure MTX and MTX-loaded niosomes in relation to the expression levels of various genes p21, p27, heat shock protein 26 (HSP26), HSP70, catalase (CAT), and superoxide dismutase 1 (SOD1) in healthy and cancerous cells by using qRT-PCR.

## 2. Materials and methods

### 2.1. Materials

MTX, Tween 80, Span 80, cholesterol, chloroform, and sodium hydroxide were purchased from Sigma–Aldrich (St. Louis, USA). All other reactive agents were of analytical grade. HAMS F 12 Eagle’s Minimum Essential Medium (EMEM) and trypsin were obtained from EDTA Multicell (Wisent Bioproducts); Dulbecco’s modified Eagle’s medium (DMEM), L-glutamine, fetal bovine serum (FBS), and penicillin–streptomycin were obtained from Gibco (Thermo Fisher Scientific); and thiazolyl blue tetrazolium bromide (MTT) was purchased from Biomatik (Ontario, Canada). Phosphate-buffered saline (PBS) and dimethyl sulfoxide (DMSO) were procured from Merck-Millipore. PureLink RNA Mini Kit was obtained from Invitrogen (Thermo Fisher Scientific); SYBR Select Master Mix and High Capacity cDNA Reverse Transcription Kit, DAPI (4′,6-Diamidino-2-Phenylindole, Dihydrochloride), CellEvent™ Caspase-3/7 (Cas 3,7) Green Detection Reagent, NucBlue™ Live ReadyProbes™ Reagent, and High-Capacity cDNA Reverse Transcription Kit were obtained from Applied Biosystems (Thermo Fisher Scientific).

### 2. 2. Preparation of niosomes

MTX-loaded niosomes were produced using the thin film method, as previously described [8, 9]. In brief, 11.25 mM of Span 80, Tween 80, and 22.5 mM of cholesterol were used in this formulation.

### 2. 3. Characterization of niosomes

Particle size (z-average), size distribution, polydispersity index (PDI), and zeta potential were determined by the DLS method by using a particle analyzer (Litesizer 500; Anton Paar, Graz, Austria) (n = 6) [10]. The indirect method was used for the quantification of the encapsulation efficiency of MTX in niosomes [8]. In the further characterization, differential scanning calorimetry (DSC), fourier transmission infrared analysis (FT-IR) and morphological analysis were carried out with our previous methods [8,11].

### 2. 4. Cell culture conditions

Human hepatocellular carcinoma (HEP3G; ATCC^®^ HB-8064™) and healthy Mus musculus hepatocyte (AML12; ATCC^®^ CRL-2254™) cells were cultured in Dulbecco’s Modified Eagle Medium (DMEM) supplemented with Eagle’s Minimum Essential Medium (EMEM), Ham’s F-12 Nutrient Mixture (HamsF-12) containing 5% of Fetal Bovine Serum (FBS) and 100 IU/mL penicillin, 10 mg/mL streptomycin and %1% L-glutamine. Cells were cultured in the oven (PANASONIC MCO-18AC-PE, Japan) at 37° C with 95% humidity and 5% CO_2_. Cells were subcultivated using trypsin EDTA (0.25%).

### 2. 5. Cell viability assay

Hepatocarcinoma cancer HEP3B cell line and AML12 normal liver cell lines were assessed to check the cytotoxic effect of MTX and MTX loaded niosome formulation by using 3-(4,5-dimethylthiazol-2-yl)-2,5-diphenyltetrazolium Bromide (MTT) assay [12, 13]. The MTT assay was performed according to the method described by Turker et al. [12]. The absorbance values were measured using a microplate reader (MultiSkan GO, Thermo Fisher Scientific Inc., Waltham, MA, USA) at 492 nm. According to the determined absorbance values, cell viability % were calculated according to the equation below:


% Cell viability: Sample absorbance value/Control absorbance value*100

### 2. 6. Immunofluorescence assay

The DAPI/CASP 3-7/ETB staining was utilized to determine the morphological alterations induced by pure MTX, MTX-loaded niosomes and blank niosomes on hepatocarcinoma (HEP3B) and healthy liver (AML12) cells. To evaluate the apoptotic activity, cancerous and healthy cells which were plated at 3 × 10^5^ cells/well into a six-well chamber plate were treated with samples and incubated for 24 h. To analyze cell death, cells were treated with IC_50_ of formulations. After 24 h of incubation, cells were washed with PBS and stained by DAPI/CASP 3-7/ETB according to manufacturer instruction. The morphological changes were investigated for apoptotic nuclei under a fluorescent microscope (Carl Zeiss, Axio Observer, Germany).

### 2. 7. Quantitation of gene expression by real-time PCR

For evaluation of gene expressions, cancerous and healthy cells were plated at 3 × 10^6^ cells/well into a six-well chamber plate. Samples at IC_50_ concentration were applied to cells. RNA isolation kit was used to separate the total RNA from pure MTX, blank niosomes, and MTX-loaded niosomes applied HEP3B and AML12 cells. The extracted total RNA was reversed to cDNA using a High-Capacity cDNA Reverse Transcription synthesis kit (Applied Biosystems, USA) according to the manufacturer’s recommendations. In the gene expression studies, qRT-PCR (Applied Biosystem, Life technology, California, US) was performed with QuantStudio™ Flex 6 Real-Time PCR System (Applied Biosystems, USA) using SYBR^®^ Select Master Mix (Life Technologies, USA) and p21, p27, CAT, SOD, HSP26, HSP70, GAPDH, Cas3, Cyc-c, Bax, Bcl-2, p53 were used as gene-specific primers. The primer sequences are shown in Table 1. Real time PCR analysis was performed using the sybr green method. Relative gene expressions were determined compared to control and normalized by Glyceraldehyde 3-phosphate dehydrogenase (GADPH) mRNA expressions. The expression levels of mRNAs were examined using the comparative cycle threshold (2-ΔΔCt) method (User Bulletin 2, Applied Biosystems, CA).

### 2. 8. Statistical analysis

SPSS (Version 20.0, Chicago, IL) was used for all statistical analyses. Mean value and standard deviation were calculated using descriptive statistics. IC_50_ values were calculated by Probit regression. In real-time PCR studies, One-way ANOVA test was used for comparison of control and experimental groups. Comparison of means was determined by Duncan Test (p < 0.0001). Significant differences were considered when p < 0.05.

## 3. Results

### 3. 1. Preparation and characterization of niosomes

**Figure 1:**
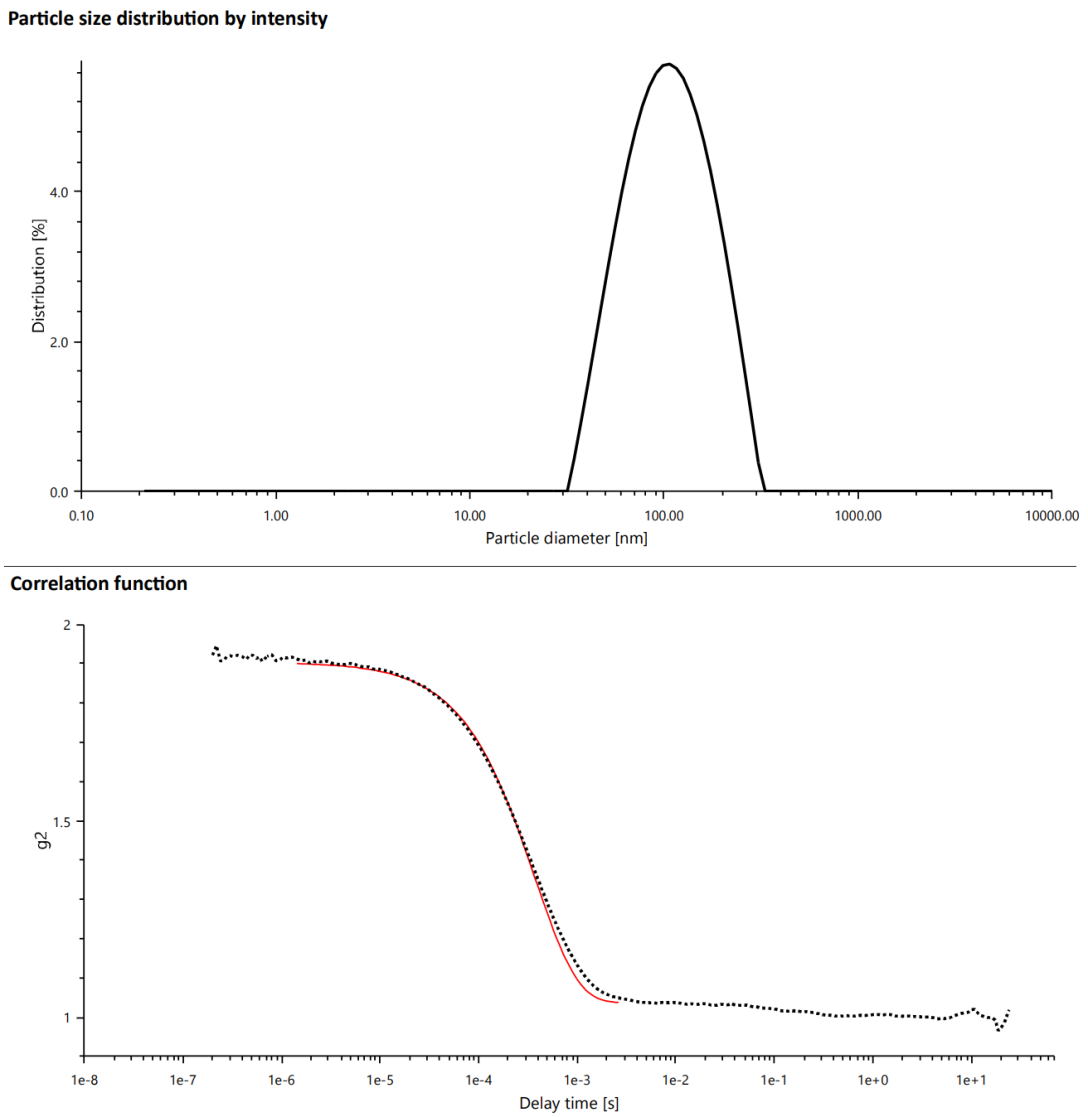
Particles size distribution of niosomes (upper) and correlation function (lower).

Niosomes were prepared through the thin-film hydration method modified with sonication. The particle size and size distribution of niosomes measured with high reliability (Figure 1 lower figure) is 117.12 ± 32.53 nm as seen in Figure 1 (upper figure).

The uniformity of the niosomal suspension was evaluated in terms of polydispersity index value which was 0.237 ± 005. The intercept of the measurements was 0.8664 and the baseline was 1.037. The zeta potential value of niosomes was –38.1 ± 0.7.

### 3.2. Interaction studies of niosomes

**Figure 2:**
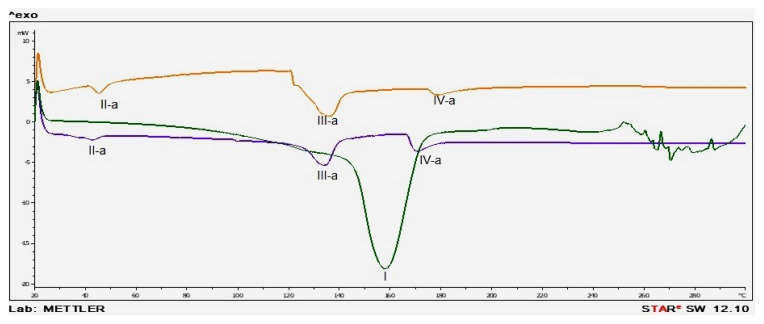
DSC thermograms of formulations (green: MTX, orange: blank niosomes, blue: MTX loaded niosomes).

**Figure 3:**
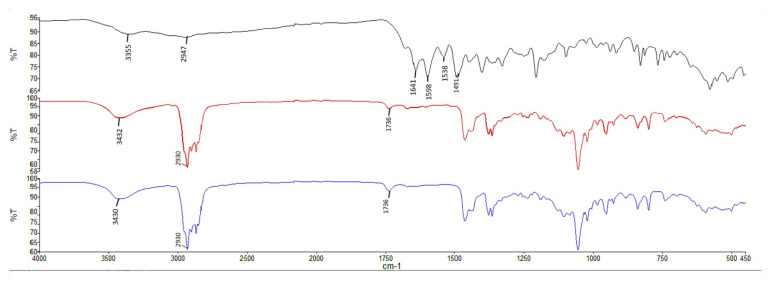
IR spectrums of formulations (black: MTX, blue: blank niosomes, red: MTX loaded niosomes).

To investigate the physical properties, such as melting points or phase transition reactions, DSC examination was conducted with MTX, blank niosome, and MTX-loaded niosomes. The DSC thermograms and FT-IR spectrums are given in Figure 2, and 3, respectively.

### 3.3. Morphology of niosomes

**Figure 4:**
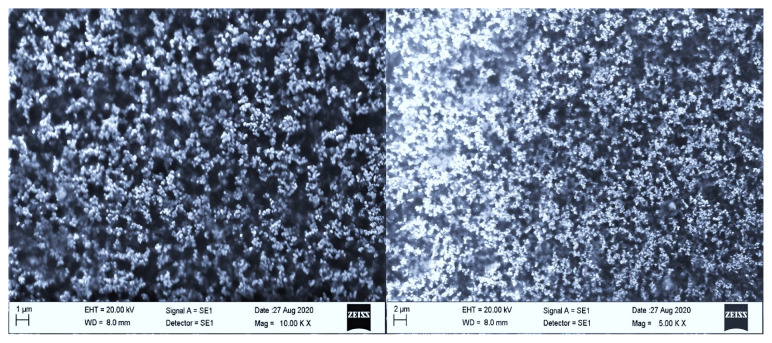
SEM images of MTX loaded niosomes; left: 5x of magnification, right: 10x of magnification.

As shown in Figure 4, the shapes of the niosomes were spherical and particle sizes varied around 100 nm.

### 3.4. Cell viability assay

**Figure 5:**
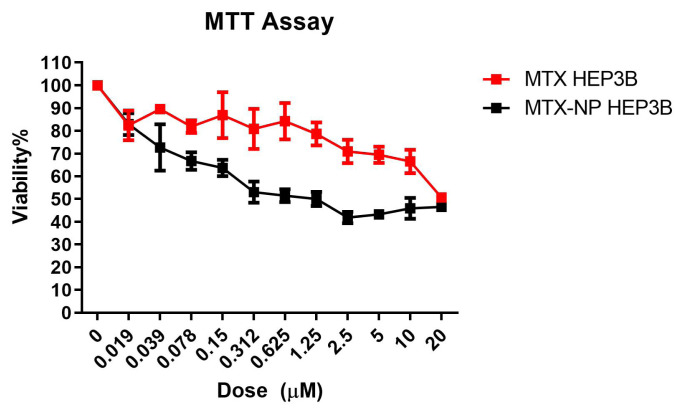
Cell viability% graph of 24-h MTX and MTX-loaded niosome applications in HEP3B cell line (n = 4 mean ± standard error), MTX-SOL refers to MTX solution whereas MTX-NIO refers to MTX loaded niosomes.

The results of the MTT assay are shown in Figure 5. Comparing cell viability results at 24^th^ and 48^th^, higher cell death was obtained at the 24^th^ h and it was selected as optimum time. MTX solution, unloaded niosomes, and MTX loaded niosomes were applied between 0.019–20 μM (dilution factor:2) to HEP3B hepatocarcinoma and AML12 cells. It was determined that there was a statistically significant decrease in HEP3B cell viability 24^th^ h after MTX solution administration (F = 36.224, p < 0.0001). The dose of IC_50_ values for MTX solution and MTX loaded niosomes were determined as 18.340 μM and 2.019 μM on the HEP3B, respectively. Moreover, the IC_50_ value was not found in the blank niosomes application within the applied dose range. These findings indicated that niosomes were clearly nontoxic on HEP3B cell lines. It was also underlined that niosomes were increased the anticancer effect of MTX overall. One of the most probable explanations was the increase of the cellular uptake through niosomes which have a similar structure to cellular membranes [14].

For AML12 cells, IC_50_ of MTX was 19.99 μM, while MTX-NIO was found above 20 μM. The IC_50_ value of MTX-NIO in HEP3B was significantly lower than in AML12. On the other hand, there were no such differences for MTX solution. Considering the IC_50_ doses; it was obvious that the MTX loaded niosome formulation was superiorly more cytotoxic than pure MTX to hepatocellular carcinoma cells (Figure 5). These satisfactory findings could be the results of two main factors at least: I) easily membrane internalization by increasing fluidity. Increasing interaction with cancer cells is one of the main benefits of colloidal vesicular particles. This cell-nanoparticle interaction could be stem from the internalization through the nonspecific phagocytosis reactions [14, 15]. II) Surfactants are used in absorption and permeability enhancement via altering fluidity, modulating tight junctions, and solubilizing cell membranes. These changes result in the increased drug retention in cells [16, 17]. Besides Tween 80s are used as an absorption enhancer by inhibiting superficial membrane P-gp proteins [18]. This approach shows that niosomal formulation improved cytotoxicity of chemotherapeutics on cancer cells and could decrease required dose and the incidence of side effects. In the previous studies, different formulation strategies were improved cytotoxicity on cancer cells. MTX loaded ultra-permeable niosomal formulations were developed by Al-Mahallawi et al., 2019. In this study, optimal niosome formulation (EE% of 65.16% and particle size of 453.6 nm) were found more cytotoxic compared to MTX solution on MCF-7 cells (IC_50-Niosome_: 98.3 μg/mL < IC_50-Solution_: 118 μg/mL) [19]. In another study, cytotoxicity of the optimum imatinib loaded niosomes formulation was evaluated on three different cancer cell lines including liver cancer (HEPG2) and a normal cell line to confirm its selectivity against cancer cells by MTT test. This niosomal imatinib showed superior efficacy on HEPG2 than those of free drug. Furthermore, the formulation’s IC_50_ against healthy cell lines was 3 to 11 times greater than that against cancer cells, indicating a stronger selectivity for the cancer cells [15]. Similar results were obtained from previous niosomal drug delivery studies [14,20].

### 3.5. qRT-PCR results

**Figure 6:**
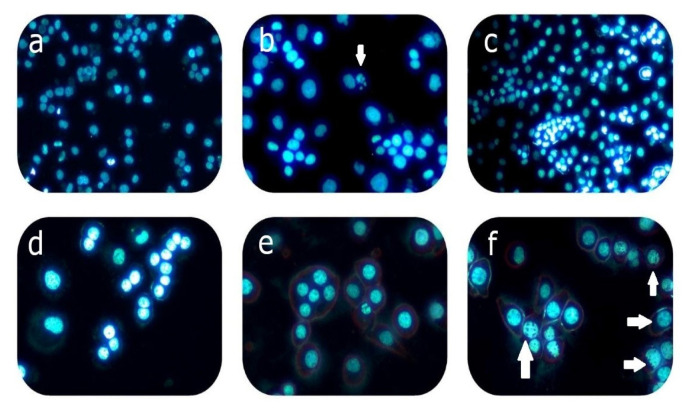
Apoptotic cells showed with arrow (^a^HEP3B control, ^b^HEP3B MTX, ^c^HEP3B MTX-NIO, ^d^AML-12 control, ^e^AML-12 MTX, ^f^AML-12 MTX-NIO).

**Figure 7:**
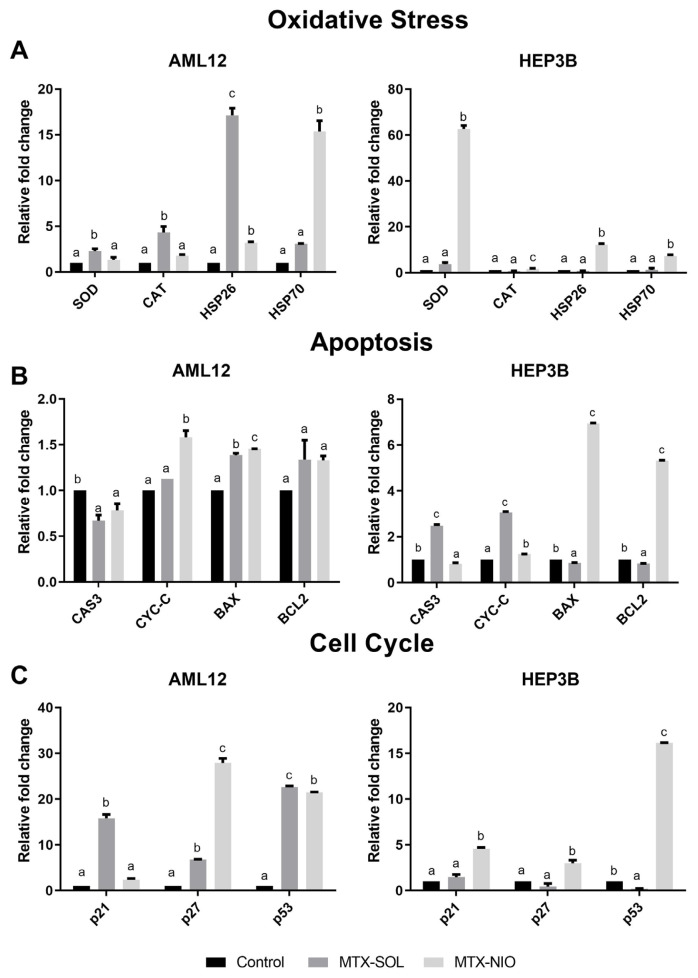
On HEP3B and AML-12 cell line, mRNA expression of; A: Oxidative Stress, B: Apoptosis and C: Cell Cycle and Signaling Pathway Genes (n: 3 ± std) (relative to the control group; one-way ANOVA post hoc Duncan, p < 0.05) (Groups indicated with different letters are statistically different from each other).

Table 2 (HEP-3B and AML-12) shows statistical (F and p values) results for all genes. Figure 6 shows photos of HEP-3B (Figures 6. a–6-c) and AML-12 cells (Figures 6. d–6-f) stained with immunofluorescence. Figure 7 shows the mRNA expression of the genes for oxidative stress, apoptosis, cell cycle signaling pathways in the HEP3B and AML-12 cell lines (n = 3).

## 4. Discussion

One of the most critical parameters of characterization on colloidal vesicular particles is the particle size that has directly an impact on cell penetration. Smaller particles exhibit increased drug absorption, as is well known. In niosome formulation, types of surfactants, surfactant composition, cholesterol concentration, and hydration solution can influence the particle sizes [8, 21, 22]. It was reported that the optimum size range is around 100–200 nm for the extent of particle uptake [23]. Our particle size result is suitable for cellular uptake. In general, PDI of 0.3 and less is suitable for lipid-based carriers in particular and denotes a homogeneous population of vesicles [24]. Moreover, according to the instruction manual for LitesizerTM 500, the ideal value of the correlation function intercept (g1^2^) should be between 0.85–0.95 and the baseline should ideally be 1.000 to get reliable measurements [25]. Findings with high reliability were stated the homogenous dispersion (Figure 1). The morphological examination also supported those results (seen in Figure 4).

The zeta potential interprets the stability of the dispersion on colloidal vesicular particles. The dispersion with high zeta potential (>30 mV, either positive or negative) withstands the aggregation, and presents high stability [23]. In other respects, the surface charge influences the cellular interaction and uptake of particles. Negatively or positively charged particles are more internalized in comparison to uncharged particles [26]. The niosomes with −38.1 ± 0.7 of zeta potential were found stable and they had great potential to be absorbed through cells.

The encapsulation efficiency is the capability of niosomes to load therapeutic agents, i.e. MTX. It is common in the range of 10% to 40%. Spans and tweens are known to have higher encapsulation efficiency, compared to other surfactants, because of the length of the alkyl chain of surfactants. The encapsulation efficiency of MTX in our study reached to 59.7% ± 2.3%. Considering the hydrophobic nature of MTX, a substantial amount of MTX (0.64 mg/mL) could be loaded into the niosomes.

According to DSC thermogram, high interaction between MTX and components could result in the shifts and disappearing peak of MTX, and this might indicate the high encapsulation of MTX in the niosomes [23, 27]. In FT-IR spectra, the fingerprint region for MTX and MTX loaded niosomes was different. Any characteristic vibrations belonging to MTX were not seen in the FT-IR analysis of MTX loaded niosomes. The results were underlined that MTX was successfully loaded into niosomes, because MTX was enclosed with niosomes and characteristic signal was disappeared.

High-dose MTX therapy for cancer treatment is known to damage the liver and kidneys. Although the exact mechanisms causing the liver and kidney damage caused by MTX need be identified, numerous investigations have demonstrated that MTX-induced cytotoxicity is linked to an increase in oxidative stress and caspase activation. In order to decrease MTX-related adverse effects and boost anticancer effectiveness, combination therapies of low-dose MTX and other anticancer medications are now being studied and used for a variety of tumor treatments [28].

Previous research looked into the mechanisms underlying sitagliptin’s hepatoprotective function against methotrexate (MTX)-induced hepatotoxicity in mice. The results indicated that MTX significantly harmed the liver, resulting in hydropic degeneration, apoptosis, and localized necrosis in all hepatic areas. Oxidative stress was also determined in hepatic tissues. In addition, an increase in the immuno-expression of the pro-apoptotic protein Bax, and caspase-3 levels; a decrease in antiapoptotic Bcl-2 level were found in the liver. Sitagliptin pretreatment significantly improved MTX-induced liver cancer [29]. Another study examined the mechanically induced cell death index in SKOV-3 ovarian cells following treatment with methotrexate (MTX), as well as the antiproliferative effect of the drug. As a result; a dose-dependent antiproliferative effect was observed in MTX treatment in SKOV-3 cells and the IC_50_ concentration was set at 40 μM MTX (p < 0.01). It has been determined that MTX increases intracellular ROS levels, causes DNA damage, increase in Bax gene, down-regulation of Bcl-2 gene expression. As a result, MTX is effective on SKOV-3 cancer cells by altering mitochondrial membrane potential with apoptotic gene up-regulation [30].

In this study different gene groups including apoptosis, antioxidant, heat shock protein and oxidative stress were evaluated. In applied MTX solution on AML-12 cells, there were 2.28- and 4.23-fold increases in the expression of oxidative stress genes especially SOD and CAT, respectively compared to control group (Figure 7a, Table 2). These increases in SOD and CAT gene expressions caused stress on AML-12 cells by disrupting the oxidative balance and cell membrane integrity/functionality. In response to this stress, the cellular defence mechanism came into effect and HSP26/HSP70 gene expressions were increased 17.12- and 3.09-fold, respectively (Figure 7b, Table 2). In this stress, p53 gene expression increased 22.62-fold. This increase showed that the E3 ubiquitin ligase complex SCF Skp2 may have induced an increase in p21 and p27 (15.80-fold, 6.79-fold, respectively) on the G1/S transition of the p53-dependent cell cycle (Figure 7c, Table 2). In the study, it was reported that after intraperitoneal injection of MTX, nucleus and nucleus fragments were stained with TUNEL staining and additionally nucleus condensation and cytoplasm condensation with apoptotic cell morphology were observed in cells in rat liver. It also showed that there was no necrosis morphology in hematoxylin-eosin staining [31]. While study reported that MTX induced apoptosis was seen in vivo, in our study MTX did not cause apoptosis but it caused DNA damage. In another study, it was reported that administration of 20 mg/kg *ip* MTX to rats caused an increase in serum AST, ALT and bilirubin levels which are biomarkers of hepatocellular damage and induced apoptosis by causing an increase BAX and caspase 3 in liver tissue. This result supported our study [32].

MTX applied to HEP3B cells caused a statistically significant decrease (0.21-fold) in tumor suppressor gene p53 expression. Bax (0.85-fold) and Bcl-2 (0.82-fold) gene expression decreased at the same rate. A statistically significant increase was observed in genes belonging to apoptosis in the HEP3B cell line especially in Cas3 (2.48-fold) and cyc-c (3.06-fold) in the MTX group. MTX may induce apoptosis independently of p53 because of the partial deletion p53 gene in HEP3B. As seen in the immunofluorescence staining images (Figure 6), bubbles called nuclear fragments formed in the last stage of apoptosis were observed. However, these changes indicated that apoptosis was triggered in a different way, not in the internal pathway. It was reported that MTX caused an increase in H_2_O_2_ in the HEP3B cell line and apoptosis with caspase-9/-3 cascade activation representing the intrinsic pathway [29]. MTX has been shown to induce apoptosis in the HEPG2 cell line. It has been also reported that MTX induces apoptosis by causing a statistically significant increase in Bax/Bcl-2 ratio in the HEPG2 hepatocarcinoma cell line [33]. It has been shown that MTX caused oxidative stress in HEPG2 cells due to ROS formation causing damage and a decrease the activity of the catalase enzymes that is the one of the antioxidant defense enzymes [34].

The pure MTX-related-stress in control group has not been observed in MTX loaded niosomes group of AML-12 cells and in this group, p53 gene increased 21.51-fold, p21 gene increased 2.38-fold which is involved in the G0/G1 phase of the cell cycle (Table 2). This caused increasement of Bax gene expression 1.45-fold and cyc-c gene expression 1.58-fold compared to the control by changing the mitochondrial structure. It leads to programmed cell death independent of oxidative stress. In the literature, no articles were found about the effect of the MTX loaded niosomes on the healthy liver cell lines or the hepatocellular carcinoma cell lines.

Application of MTX loaded niosome to the HEP3B cell line increased the tumor suppressor gene p53 gene expression, but it could not realize its function due to the partial deletion of this gene in the HEP3B cell line. Also, Bax expression increased 6.93-fold compared to the control (Table 2). It was determined that the application of MTX loaded niosomes to HEP3B cells caused two different cellular signal transductions based on qRT-PCR results. In previous research, it was shown that MTX loaded nanocomposites caused a significant decrease in the percentage of living cells with a significant down-regulation of the antiapoptotic gene (Bcl-2) and up-regulation of the pro-apoptotic gene expressions (Bax). Besides, down-regulation of Bcl-2 stimulates oxidative stress. Increased Bax/Bcl-2 ratio by nanocomposites showed effect of nanocomposites in the inhibition of cell proliferation and triggering of apoptosis [35]. In our study, especially the increase in Bax protein (6.93-fold) changed the membrane potential of mitochondria and increased the cyc-c gene that passes into cytosol 1.24-fold in the MTX loaded niosome group of HEP3B cells (Figure 7b, Table 2). Bcl-2 gene expression increased approximately 5.32-fold. Impaired Bax/Bcl-2 ratio leaded apoptosis inhibition and suppressed mitochondrial apoptosis pathway. Bax/Bcl-2 ratio changes onset of apoptosis. However, in especially cyc-c (1.24-fold) levels were found compared to the control; there were 4.56- and 2.98-fold increases in p21^cip1^ and p27^kip1^, respectively. This increase should stop the cell cycle. The statistically significant increases were seen in p21^cip1^ and p27^kip1^ arrest genes which indicated that cell death may be caused by apoptosis. However, no apoptotic cells were found in the immunofluorescence staining images (Figure 6). Considering the increases, cell death might be induced through any pathway other than the mitochondrial pathway of apoptosis. According to the oxidative stress genes; SOD gene expressions in MTX loaded niosome treated cells increased 62.71-fold compared to the control causing oxidative stress. This increase is thought to impair cell membrane integrity and function. Increased HSP70 gene expression (7.27-fold) showed that invasive and metastatic features of HEP3B cells were suppressed (Figure 7a, Table 2).

Based on all results, oxidative stress-induced DNA damage in the MTX group in AML-12 cells initiated apoptotic cell death with MTX-NIO administration. The results showed that niosomal structure can facilitate passage of MTX to reach the target tissues.

Our results showed that the use of MTX-loaded niosomes in therapy can prevent oxidative stress caused by MTX toxicity. Supporting our findings with in vivo studies will increase the clinical significance of MTX-loaded niosomes.

Hepatotoxicity of MTX is a common complication of long-term treatment with MTX. Minimizing these complications is crucial for this reason. For this aim, we developed MTX loaded niosomes and investigate their hepatotoxicity compared to MTX solution. MTX loaded niosomes were prepared by modified thin film method successfully. No characteristic peaks in IR spectrum and thermogram proved the absence of interaction. Therefore, the results pointed that MTX was dispersed well in niosomal formulation. In cytotoxicity study, MTX-loaded niosomes showed higher effect on cancer cells than healthy cells. Our results showed that the use of MTX-loaded niosomes in MTX-therapy can prevent oxidative stress caused by MTX toxicity. Supporting our findings with in vivo studies will increase the clinical significance of MTX-loaded niosomes.

## Figures and Tables

**Table 1:** Primer sequences.

Gene	Sequences
BCL-2	F: 5′-ATGTGTGTGGAGAGCGTCAA-3′ R: 5′-ACAGTTCCACAAAGGCATCC-3′
Bax	F: 5′-TTCATCCAGGATCGAGCAGA-3′ R: 5′-GCAAAGTAGAAGGCAACG-3′
CYC-C	F: 5′-AGTGGCTAGAGTGGTCATTCATTTAC-3′ R: 5′-TCATGATCTGAATTCTGGTGTATGAG-3′
GAPDH	F: 5′-AATTCCGATCTTCGACATGG-3′ R: 5′-GAAAAAGCGGCAGTCGTAAT-3′
CASP 3	F: 5′-GGTATTGAGACAGACAGTGG-3′ R: 5′-CATGGGATCTGTTTCTTTGC-3′
p21	F: 5′-GGCGTTTGGAGTGGTAGAAA-3′ R: 5′-GACTCTCAGGGTCGAAAACG-3′
p27	F: 5′-CCGGCTAACTCTGAGGACAC-3′ R: 5′-TGGATCCAAGGCTCTAGGTG-3′
p53	F: 5′-CACGAGCGCTGCTCAGATAGC-3′ R: 5′-ACAGGCACAAACACGCACAAA-3′
CAT	F: 5′-TACGAGCAGGCCAAGAAGTT-3′ R: 5′-ACCTTGTACGGGCAGTTCAC-3′
SOD	F: 5′-GTTCGGTGACAACACCAATG-3′ R: 5′-GGAGTCGGTGATGTTGACCT-3′
HSP26	F: 5′-CAAGCAGCTGAACAAGCTAACAATCTG-3′ R: 5′-GCATGATGTGACCATGGTCGTCCTGG-3′
HSP70	F: 5′-GAACGGGCCAAGCGCACACTCTC-3′ R: 5′-TCCTGGATCTTGCCGCTCTGGTCTC-3′

**Table 2:** Anova/Duncan test results (F and p values) of all studied genes (HEP-3B and AML-12).

	HEP3B					AML-12				
GEN ID	Control	MTX-Sol	MTX-NIO	F	P	Control	MTX-Sol	MTX-NIO	F	P
SOD	1.005	3.791	62.717	1413.580	.000	1.000	2.283	14.410	9.698	.013
CAT	1.037	0.712	1.653	9.256	.015	1.015	4.324	1.917	20.486	.002
HSP70	1.019	1.191	7.279	39.588	.000	1.004	3.092	12.702	131.977	.000
P27	1.034	0.442	2.989	23.798	.001	1.010	6.799	4.314	625.148	.000
P21	1.031	1.470	4.564	105.951	.000	1.069	15.805	7.132	250.637	.000
HSP26	1.028	0.842	12.259	661.014	.000	1.004	17.120	6.404	366.236	.000
CAS3	1.024	2.483	0.823	437.698	.000	1.070	2.484	0.073	9.668	.013
CYC-C	1.021	3.063	1.250	765.758	.000	1.096	3.061	1.850	53.338	.000
BAX	1.018	0.860	6.933	5126.900	.000	1.045	0.858	1.941	385.401	.000
BCL2	1.014	0.823	5.323	2055.983	.000	1.000	0.827	0.243	2.339	.177
P53	1.011	0.213	16.163	18992.431	.000	1.008	0.215	0.370	6956.966	.000
